# Alcohol control policies reduce all-cause mortality in Baltic Countries and Poland between 2001 and 2020

**DOI:** 10.1038/s41598-023-32926-5

**Published:** 2023-04-18

**Authors:** Justina Vaitkevičiūtė, Inese Gobiņa, Kinga Janik-Koncewicz, Shannon Lange, Laura Miščikienė, Janina Petkevičienė, Ričardas Radišauskas, Rainer Reile, Mindaugas Štelemėkas, Relika Stoppel, Tadas Telksnys, Alexander Tran, Jürgen Rehm, Witold A. Zatoński, Huan Jiang

**Affiliations:** 1 Health Research Institute, Faculty of Public Health, Lithuanian University of Health Sciences, Tilžės str. 18, 47181 Kaunas, Lithuania; 2 Department of Public Health and Epidemiology, Riga Stradiņš University, Kronvalda Boulevard 9, Riga, 1010 Latvia; 3 Institute of Public Health, Riga Stradiņš University, Kronvalda Boulevard 9, Riga, 1010 Latvia; 4 European Observatory of Health Inequalities, Calisia University, Nowy Swiat 4, 62-800 Kalisz, Poland; 5 Health Promotion Foundation, Mszczonowska 51, 05-830 Nadarzyn, Poland; 6 Institute for Mental Health Policy Research, Centre for Addiction and Mental Health, 33 Ursula Franklin Street, Toronto, ON M5S 2S1 Canada; 7 Campbell Family Mental Health Research Institute, Centre for Addiction and Mental Health, 250 College St., Toronto, ON M5T 1R8 Canada; 8 Department of Psychiatry, University of Toronto, 250 College Street, 8th floor, Toronto, ON M5T 1R8 Canada; 9 Department of Health Management, Faculty of Public Health, Lithuanian University of Health Sciences, Tilžės str. 18, 47181 Kaunas, Lithuania; 10 Department of Preventive Medicine, Faculty of Public Health, Lithuanian University of Health Sciences, Tilžės str. 18, 47181 Kaunas, Lithuania; 11 Department of Environmental and Occupational Medicine, Faculty of Public Health, Lithuanian University of Health Sciences, Tilžės str. 18, 47181 Kaunas, Lithuania; 12 Institute of Cardiology, Lithuanian University of Health Sciences, Sukilėlių av. 15, 50162 Kaunas, Lithuania; 13 Department for Epidemiology and Biostatistics, National Institute for Health Development, Hiiu 42, 11619 Tallinn, Estonia; 14 Department of Economics, University of Potsdam, August-Bebel-Straße 89, 14482 Potsdam, Germany; 15 Dalla Lana School of Public Health, University of Toronto, 155 College Street, Toronto, ON M5T 1P8 Canada; 16 World Health Organization/Pan American Health Organization Collaborating Centre, Centre for Addiction and Mental Health, 33 Ursula Franklin Street, Toronto, ON M5S 2S1 Canada; 17 Institute of Clinical Psychology and Psychotherapy, Center of Clinical Epidemiology and Longitudinal Studies (CELOS), Technische Universität Dresden, Chemnitzer Str. 46, 01187 Dresden, Germany; 18 Center for Interdisciplinary Addiction Research (ZIS), Department of Psychiatry and Psychotherapy, University Medical Center Hamburg-Eppendorf (UKE), Martinistraße 52, 20246 Hamburg, Germany; 19 Faculty of Medicine, Institute of Medical Science, University of Toronto, Medical Sciences Building, 1 King’s College Circle, Room 2374, Toronto, ON M5S 1A8 Canada; 20 Program on Substance Abuse, Public Health Agency of Catalonia, Program on Substance Abuse & designated WHO CC, Public Health Agency of Catalonia, 81-95 Roc Boronat St., 08005 Barcelona, Spain

**Keywords:** Epidemiology, Disease prevention, Health policy, Public health

## Abstract

Alcohol consumption in the Baltic countries and Poland is among the highest globally, causing high all-cause mortality rates. Contrary to Poland, the Baltic countries have adopted many alcohol control policies, including the World Health Organization (WHO) “best buys”. The aim of this study was to evaluate the impact of these policies, which were implemented between 2001 and 2020, on all-cause mortality. Monthly mortality data for men and women aged 20+ years of age in Estonia, Latvia, Lithuania, and Poland were analysed for 2001 to 2020. A total of 19 alcohol control policies, fulfilling an a-priori defined definition, were implemented between 2001 and 2020 in the countries of interest, and 18 of them could be tested. Interrupted time-series analyses were conducted by employing a generalized additive mixed model (GAMM) for men and women separately. The age-standardized all-cause mortality rate was lowest in Poland and highest in Latvia and had decreased in all countries over the time period. Taxation increases and availability restrictions had short-term effects in all countries, on average reducing the age-standardized all-cause mortality rate among men significantly (a reduction of 2.31% (95% CI 0.71%, 3.93%; *p* = 0.0045)). All-cause mortality rates among women were not significantly reduced (a reduction of 1.09% (95% CI − 0.02%, 2.20%; *p* = 0.0554)). In conclusion, the alcohol control policies implemented between 2001 and 2020 reduced all-cause mortality among men 20+ years of age in Baltic countries and Poland, and thus, the practice should be continued.

## Introduction

Alcohol is one of the main risk factors for non-communicable disease and injury, with more than 200 health conditions causally impacted^1,2^. In the World Health Organization (WHO) European Region, 10.1% of all deaths were attributable to alcohol use in 2016^2^. Alcohol-attributable fractions (AAF) are the highest in the WHO European Region, compared to other WHO regions^3^. AAFs were especially high in Central, Eastern Europe and Baltic Countries^4^.

However, alcohol-attributable deaths and all-cause mortality could be reduced and life expectancy increased through the implementation of effective alcohol control policies^5,6^. Three policies have been highlighted by the WHO to be especially effective, cost-effective, and easy to implement; and have been labelled as “best-buys”. These measures include increasing taxes on alcoholic beverages, enacting and enforcing bans or comprehensive restrictions on exposure to alcohol advertising across multiple types of media, and enacting and enforcing restrictions on the physical availability of alcoholic beverages, for example, via reduced hours of sale^7^. Recent evidence corroborated this recommendation^8^. However, even though alcohol tax policies have been proven to be an effective tool in reducing alcohol harm, and there was strong evidence demonstrating the benefits of alcohol control fiscal policies^9–11^, they have been generally under-implemented in the European region^4,12^.

When evaluating the effectiveness of alcohol control policies, it is important to consider the influence of other factors related to alcohol consumption, such as changes in alcohol affordability due to the economic situation, inflation, purchasing power, growth of salaries and gross domestic product (GDP). For example, the economic crisis, which started in 2008, may have had an impact on the population's alcohol consumption due to the decline in income^13^.

The three Baltic countries implemented different levels of alcohol availability, affordability, and marketing restrictions in the past 20 years^14,15^, meanwhile Poland established only a taxation increase in 2020, and even loosened control in 2002 via a decrease in alcohol excise taxation^15^. Some of these effects have been evaluated such as the 2017 alcohol control policy of increased excise taxation implemented in Lithuania^11,16^. However, a single evaluation of an alcohol control policy is subject to potential confounding by other events and conditions which may have happened at the same point in time^17^. Thus, we need to introduce as much control as possible; analysing several interventions in different countries in the same region allows for such control, and aids in determining the general impact of policies by aggregating effects across various time points and conditions. As the Baltic countries and Poland differ in the strictness of implemented alcohol control policies and the time points when the policies were adopted, they constitute unique conditions to evaluate such effects, where the other countries can serve as control conditions.

Concretely, we identified all policies that had been implemented within a timespan of two decades, which fulfilled clear a priori defined criteria of decreased affordability and availability [see^15^]. Simultaneously measuring their average effect allowed for: (a) more generalizable estimates on average effect sizes of such policies; (b) better control for secular trends across the region, since we can use the other countries as control conditions for each policy effect; (c) better control of all other factors, as it is highly unlikely that, at exactly the same time of all 18 alcohol control interventions other events occurred which triggered the effects.

Thus, the aim of this study was to evaluate the impact of alcohol control policies applied by Poland and the three Baltic countries—Estonia, Latvia, and Lithuania—on all-cause mortality rates among the adult population (20+ years of age) between 2001 and 2020. All-cause mortality was selected as the main outcome rather than alcohol-attributable mortality, as it is the most important endpoint from a public health point of view. Even though, this endpoint appeared only once in the meta-analyses of Wagenaar and colleagues^18^, it has been shown that in high-consuming countries of the WHO European Region, it can be affected by effective alcohol-control policies (e.g.,^5,6,11^). To ascertain, that the effect was indeed based on causes of death related to alcohol we conducted a decomposition based on data from Lithuania and Estonia, where we had cause-specific data. Concretely, we hypothesized that the alcohol control policies implemented reduced all-cause mortality in the Baltic countries and Poland in 2001–2020 in the adult population.

## Methods

### Data

The data for 2001–2020 (with exception of Poland, for which data covered the years 2001–2019) was obtained for Estonia from Statistics Estonia^19^, for Latvia from the Official Statistics of Latvia^20^, for Lithuania from Statistics Lithuania^21^ and The State Register of Death Cases and Their Causes^22^ and for Poland from the National Statistical Office^23^. To avoid including the impact of the coronavirus 2019 pandemic, data for December 2020 were excluded from the analysis, given its high impact in this month, and the fact that it was the last month of the series, which should not impact on the overall evaluation. Population data were used to convert mortality count data to rates were obtained for each country from the Organisation for Economic Co-operation and Development (OECD)^24^. Mortality rates were standardized according to EU standard^25^.

### Outcomes

The dependent variable was monthly age-standardized all-cause mortality rate per 100,000 population for individuals 20+ years of age for men and women. A decomposition of changes by cause of death was undertaken for Lithuania and Estonia, as we had obtained cause of death data for these two countries. Deaths were calculated 12 months before (“pre-policy”) and 12 months after (“post-policy”) the implementation of the policy by country and sex (and summed up over all the policies in the respective country for Table 2, details in Supplementary Table S7).

### Intervention (alcohol control policies)

A total of 18 policies, classified as “best buys” and expected to have an immediate effect, were applied in the study period based on the following criteria^15^: (1) taxation increases should decrease affordability of alcoholic beverages (affordability was measured by data on alcohol prices, inflation and disposable income); and (2) availability restrictions should reduce availability by at least 20% (decrease in opening hours at least 20%). Prior analyses showed that these policies had an impact on consumption level^26^. In Estonia, eight policies were selected; Latvia, five policies; Lithuania four policies; and in Poland, one policy (see Table 1; for detailed description of policies see Supplementary Tables S1–S4). The policy implemented in Poland could not be tested due to a lack of data for 2020 for this country. The effect was modelled to last for one year after implementation, and to test, whether other events at the same time in the country of implementation were impacting on the effect of the policy, we tested interaction terms between the countries and policies. One year was chosen as the time reference, as inflation and disposable income were expected to have an impact on affordability to diminish the impact of tax increases, and as we expected people to develop coping strategies to deal with availability restrictions. However, we tested a sensitivity analysis with a longer lasting but diminishing impact of the policies. For this sensitivity analyses, we set the full effect at 100%, and then reduced it by 10% per year in the following years (see Supplementary Table S6).

**Table 1:** Summary of alcohol control policies implemented in Baltic countries and Poland 2001–2020.

Year	Estonia	Latvia	Lithuania	Poland
2002		*June 14* Availability reduced		
2008	*January 1* Taxation increase, *July 1* Taxation increase, *July 14* Availability reduced	*February 1, July 1* Taxation increase	*January 1* Taxation increase, Marketing restricted	
2009			*January 1* Availability reduced	*March 1* Taxation increase
2010	*January 1* Taxation increase	*February 1* Taxation increase		
2016	*February 1* Taxation increase			
2017	*February 1*, *July 1* Taxation increase		*March 1* Taxation increase	
2018	*February 1* Taxation increase		*January 1* Availability reduced, Marketing restricted	
2019		*March 1* Taxation increase		
2020				*January 1* Taxation increase*

*This policy was not included in data analysis, because 2001–2019 Poland data were used. More details about all policies published before^14,15,26^.

### Potential confounding variables

The effect of intervention was adjusted by the effects for level of mortality in countries and economic recession on mortality. The latter was defined by a decrease in GDP based on purchasing power parities (GDP-PPP) based on data from OECD^24^. This operationalization was country-specific, as the recession of 2008 affected the countries studied differently (see Supplementary Table S5).

### Statistical analyses

To test our hypothesis that alcohol control policies led to a reduction in all-cause mortality in the Baltic countries and Poland, we performed interrupted time-series analyses by employing a generalized additive mixed model (GAMM) for both men and women^27^. Both GAMM models controlled for the economic recession using a dummy coded variable, coded as 1 during the months which were associated with the recession, and 0 for all other time points. All four countries were included in the analyses and represented by a categorical variable, with Poland as the reference category. That is, the coefficients of country effects could be interpreted with respect to Poland. The log-transformed standardized all-cause mortality rates were approximately normally distributed, allowing for the use of linear models, and easy transformation into percentage change^28^. Seasonality was adjusted for by adding smoothing splines representing monthly and yearly patterns. Residuals were examined with plots of the autocorrelation function and the partial autocorrelation function to determine the orders of autoregressive and moving average series.

For both men and women, we presented a full model and a reduced model. In the full model, the linear time trend and policy effects were investigated, adjusted by economic recession, the countries and the interactions between policies and countries, in addition to the smooth terms. Akaike information criterion (AIC) and R-squared were used to assist with selecting the most appropriate model^29^. A lower AIC value indicates a better fit; as such, the model with the lowest AIC was selected. The reduced model was created by optimizing the full model, which involved removing any non-significant covariates that did not improve the model’s fit. At last, Chi-square difference tests were used to evaluate if the full model fits significantly better or worse than the reduced model^30^. All analyses were performed using R version 3.6.3^31^.

Diagnostic graphs for the full and reduced models for both men and women can be found as Supplementary Figs. S1–S4.

## Results

**Figure 1:**
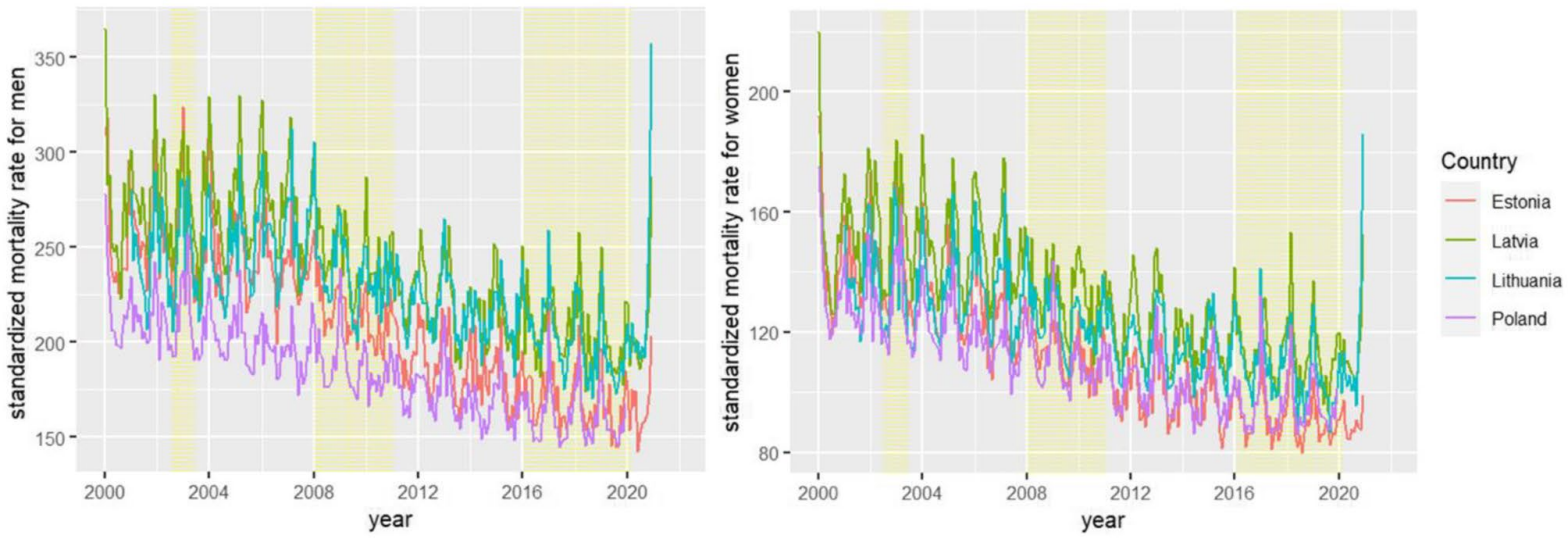
Standardized all-cause mortality rates (per 100,000) across time: the yellow shadow represents the occurrence of policy interventions.

A total of 240 months of data were included in the analyses. Figure 1 shows the standardized mortality rates over time, which show a downward trend and evidence of seasonal variation. For both men and women, GAMM confirmed that the standardized mortality rates decreased over time after adjustment (see Tables 2 and 3).

**Table 2:** Model statistics for the effects of the alcohol policies on age-standardized all-cause mortality rate for men.

	Full model				Reduced model			
	Estimate	Std. Error	95% CI	Pr( >|t|)	Estimate	Std. Error	95% CI	Pr( >|t|)
(Intercept)	5.365	0.013	(5.34, 5.39)	< 0.001	5.387	0.014	(5.359, 5.415)	< 0.001
Time (months)	− 0.001	0.000	(− 0.001, − 0.001)	< 0.001	− 0.001	0.000	(− 0.002, − 0.001)	< 0.001
Estonia	0.149	0.018	(0.114, 0.183)	< 0.001	0.130	0.010	(0.11, 0.149)	< 0.001
Latvia	0.328	0.015	(0.299, 0.357)	< 0.001	0.269	0.009	(0.251, 0.287)	< 0.001
Lithuania	0.241	0.011	(0.219, 0.263)	< 0.001	0.228	0.008	(0.213, 0.244)	< 0.001
Policy Intervention	− 0.024	0.016	(− 0.055, 0.008)	0.145	− 0.023	0.008	(− 0.038, − 0.007)	0.005
Recession	0.000	0.009	(− 0.017, 0.016)	0.978				
Estonia*policies	0.013	0.018	(− 0.022, 0.047)	0.478				
Latvia*policies	0.007	0.018	(− 0.028, 0.042)	0.694				
Lithuania*policies	0.006	0.018	(− 0.03, 0.041)	0.748				
	AIC = − 2787				AIC = − 2695

**Table 3:** Model statistics for the effects of the alcohol policies on age-standardized all-cause mortality rate for women.

	Full model				Reduced model			
	Estimate	Std. Error	95% CI	Pr( >|t|)	Estimate	Std. Error	95% CI	Pr( >|t|)
(Intercept)	4.904	0.016	(4.874, 4.934)	< 0.001	4.904	0.015	(4.874, 4.934)	< 0.001
Time (months)	− 0.001	0.000	(− 0.001, − 0.001)	< 0.001	− 0.001	0.000	(− 0.001, − 0.001)	< 0.001
Estonia	0.194	0.012	(0.171, 0.216)	< 0.001	0.194	0.011	(0.172, 0.215)	< 0.001
Latvia	0.086	0.015	(0.057, 0.114)	< 0.001	0.086	0.014	(0.058, 0.114)	< 0.001
Lithuania	− 0.047	0.017	(− 0.081, − 0.013)	0.007	− 0.047	0.017	(− 0.081, − 0.014)	0.006
Policy Intervention	− 0.010	0.009	(− 0.027, 0.008)	0.295	− 0.011	0.006	(− 0.022, 0)	0.055
Recession	− 0.008	0.009	(− 0.026, 0.01)	0.384				
Estonia*policies	− 0.002	0.013	(− 0.027, 0.023)	0.875				
Latvia*policies	0.004	0.013	(− 0.021, 0.029)	0.769				
Lithuania*policies	− 0.013	0.019	(− 0.05, 0.025)	0.509				
	AIC = − 2739				AIC = − 2745			

### Effects of policy

The policy effects estimated in the full and reduced models were similar (Table 2). For example, the estimate for the policy effect for men was − 0.024 in the full model compared to − 0.023 in the reduced model (Table 2), despite the fact that standard errors were larger in the full model due to more variables included. In the final reduced model, alcohol control policies had a significant effect on the all-cause mortality rate among men (0.0228 (95% CI 0.0071, 0.0385; p = 0.0045)), which transformed into a reduction of 2.31% (1− exp(0.0228)*100%, 95% CI 0.71%, 3.93%) in all-cause mortality rate (Table 2). Given the different numbers of deaths in the countries, this corresponds to average effects of about 172, 317, 478, and 4340 deaths avoided per year for Estonia, Latvia, Lithuania, and Poland, respectively.

For females, the effect was not significant (0.0108; 95% CI − 0.0002, 0.0218; p = 0.0554), however because it approached a p < 0.05 threshold, we computed its effect, which transformed into 1.09% (1− exp(0.0108)*100%, 95% CI − 0.02%, 2.20%), corresponding to average effects of around 84, 159, 218, and 1892 deaths avoided per year for Estonia, Latvia, Lithuania, and Poland, respectively (Table 3).

### Effects of countries

For men, Latvia had a higher age-standardized all-cause mortality rate than Poland (by 38.82% = exp(0.328) − 1)) (Table 2). Estonia and Lithuania also showed higher mortality than Poland: Estonia by 16.01% (exp(0.1485) − 1) and Lithuania by 27.26% (exp(0.2411) − 1). The interaction effects of countries with alcohol control policies in the full model were not statistically significant, which reflected that the policies did not change all-cause mortality rates differently among the four countries.

For women, compared with Poland, Estonia and Latvia also had higher age-standardized all-cause mortality rate: Estonia by 21.40% (exp(0.1939) − 1) and Latvia by 8.93% (exp(0.0855) − 1) (Table 3). Lithuania, on the other hand, had a significantly lower age-standardized all-cause mortality rate, by − 4.57% (exp(− 0.0468) − 1) on average. Similar to men, the interaction effects between countries and policies in the full model were not statistically significant.

**Figure 2:**
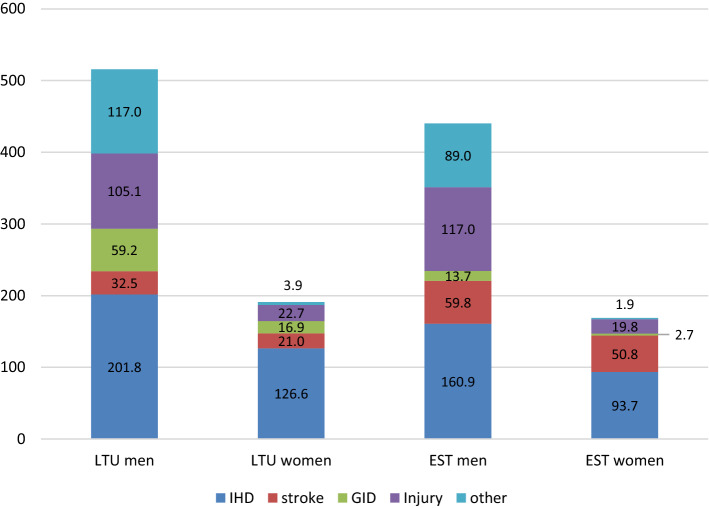
Mortality gains in age-standardized all-cause mortality rates per 100,000 population in the 11 interventions in Lithuania and Estonia, by broad causes of death. *LTU* Lithuanian, *EST* Estonian, *IHD* ischemic heart disease, *GID* gastrointestinal diseases.

Figure 2 shows the mortality gains summed up over the 11 interventions in total and by broad causes of death. Overall, men showed markedly more gains than women. Differentiating by cause of death, ischemic heart disease had the largest mortality gains in mortality followed by injury, stroke and gastrointestinal disease. All of these categories are causally linked to alcohol^1^, and all of them are expected to change abruptly with changes in exposure, as evidenced by the changes in mortality due to the Gorbachev reforms^32^. As expected, there had been only minor changes in cancer mortality, in total comprising less than 5% of the total decrease (see Supplementary Table S7). While certain cancers have been identified as causally impacted by alcohol use, this relationship has a long lag-time, so no immediate changes of the alcohol control measures were expected^32^.

To test the impact of the assumption of an impact lasting one year, a sensitivity analysis was performed assuming that policies have longer lasting effects: after the first year, the effect decreased by 10% each year to account for inflation, higher disposable income, and adaptation to availability restrictions. With this assumption, the effect for policy was no longer significant (details in the Supplementary Table S6). While the overall policy was not significant anymore, the policy in Estonia and Lithuania did show significant reductions in mortality.

## Discussion

The alcohol control policies implemented in the Baltic countries and Poland were effective in significantly reducing all-cause mortality among men. Given the difference in the number of deaths across countries, the reduction corresponds to average effects of about 172, 317, 478, and 4340 deaths avoided per year for Estonia, Latvia, Lithuania, and Poland, respectively. Analyses by cause of death in a subsample confirmed that the reduction was indeed based on alcohol-attributable causes of death.

With respect to all-cause mortality among women, the impact of implemented alcohol control policies was in the right hypothesized direction (i.e., a reduction); however, it was not statistically significant. As such, the results of the data analysis partially confirm our hypothesis.

The results of this study contribute to other previous studies showing the effectiveness of alcohol control policies in reducing all-cause mortality^6,11^, when the WHO “best buys” alcohol control policies were adopted^7^.

There are several explanations for the differential impact on men and women. Men are more likely than women to consume alcohol, they consume more alcohol when they consume, they have a higher prevalence of alcohol use disorders, and they are more prone to behavioural problems associated with acute alcohol consumption^33^although women have a shorter time period between initiation of regular alcohol use and problem use compared to men^34^ and the gap between need and receipt of treatment is larger for women than for men^35^. In the WHO European Region the consumption of pure alcohol was four times higher among men compared to women in 2016, as a result the proportion of alcohol-attributable deaths was 12.3% among men and 7.8% among women^4^. It is possible that the stronger relationship between policies and all-cause mortality in men compared with women may be because alcohol accounts for a larger proportion of total mortality in men than in women. Although alcohol control policies of taxation increase and availability reduction in the Baltic countries and Poland have been found to be effective in reducing alcohol per capita consumption of pure alcohol per year^26^, future evaluation of the impact of these policies on specific population groups such as men and women would be important.

Life expectancy is determined by many factors, including demographic, economic, social, health system, and environmental factors. Also, health behaviours (like alcohol consumption) could have an impact on the average life expectancy of a country's population^36^. The average life expectancy of men and women also varied between the Baltic countries and Poland, the inequalities being particularly large in Baltic countries, for instance in 2019 the average life expectancy difference between men and women in Lithuania was 9.6 years (men—71.6 years, women—81.2 years), for comparison in Poland difference was 7.8 (men—74,1 years, women—81.9 years)^37^. As Stumbrys and colleagues indicated, positive changes in Lithuanian’s men life expectancy in 2007–2017 were result of decreased mortality from external causes of death, cardiovascular diseases and alcohol-related disorders^38^. These causes are clearly related to alcohol consumption, and therefore alcohol control policies are expected to have an impact on them. Changes in women's mortality were less related to alcohol consumption and mortality from external causes of death, therefore alcohol control policies had less influence on them^38^. As a result, due to alcohol control policies implemented in Lithuania between 2008 and 2018, men’s age-standardized mortality decrease was higher compared to women^11^. On the contrary, increase in alcohol consumption in Poland between 2002 and 2019 was found to be a feasible cause of slow down and then halted increase of life expectancy^39^.

We would like to point out potential limitations. All-cause mortality could be affected by various factors that is difficult to identify and control (prevention programs, health care funding, economic crisis, other policies, coronavirus 2019 pandemic, etc.). These factors were not controlled in this study. Therefore, we can assess the associations of policy and mortality indicators but cannot specifically imply on causality. However, in order to give alternative explanations of the results, these factors must have occurred exactly at the same months as the alcohol control policies in each or at least most of the 18 instances, which is unlikely. While we controlled for interactions between country and policies, we did not control for three-way interactions (i.e., time, country, policy). Also, we relied on the assessment that implementation of the taxation increases which reduced affordability and availability restrictions had similar effect sizes. This assumption was based on expert judgement only^15^. Therefore, different policies and the different number of policies included in the analysis could have an impact on cross-country comparisons. Finally, we modelled the effect of alcohol control policies to last one year. While the effects of such policies clearly will diminish due to factors such as inflation, increases in disposable income for taxation, and adaptation of consumers for availability restrictions (e.g.,^40^), the 1-year assumption is likely an underestimate. However, a sensitivity analysis with longer lasting effects, showed different effects by country, with the effects only being significant in Lithuania and Estonia.

Against our hypotheses, the economic crisis of 2008 which affected the Baltic countries at slightly different times (see above) contributed to a reduction of affordability of alcoholic beverages and was associated with a reduction of alcohol per capita consumption^26^. However, within an economic crisis or other crisis situation there may also be an increase in heavier drinkers despite the overall volume of alcohol going down^13,41–43^. Economic crisis has affected funding for the health systems by decreasing public expenditures on health care, which may have affected health indicators (life expectancy, all-cause mortality etc.) and public health was particularly affected in countries with highest recession^44^.

It is important to mention that uniqueness of this study is that it evaluates the impact of immediately acting “best buy” alcohol control policies implemented in Estonia, Latvia, and Lithuania, on a broad measure, all-cause mortality, without distinguishing between policies and countries. Poland was included in the analyses, but as a control country only, as the only alcohol control policies that met the a priori defined criteria implemented between 2000 and 2020 was outside the range of data available for analyses. Since we included all instances of policy change, and still found impact on all-cause mortality, this indicates, that the “best buy” policies of taxation increases, and availability restrictions are still very valid and should be used more often to decrease all-cause mortality.

This study has demonstrated that alcohol control policies were associated with a reduction in all-cause mortality among men in the Baltic countries between 2001 and 2020. All-cause mortality among women also decreased, but not significantly so.

## Supplementary Information

Supplementary Information.

## Data Availability

Mortality data of the countries is held by government institutions and is not publicly available. Data can be provided by the responsible government institutions of the countries upon request (National Statistical Office of Poland, Official Statistics of Latvia, Statistics Estonia, Statistics Lithuania and The State Register of Death Cases and Their Causes, Lithuania).

## References

[1] Rehm J (2017). The relationship between different dimensions of alcohol use and the burden of disease—An update. Addiction.

[2] World Health Organization. *Global status report on alcohol and health 2018.* http://www.who.int/substance_abuse/publications/global_alcohol_report/en/ (2018).

[3] World Health Organization. Regional prevalence, AAFs, all-cause deaths (%). *The Global Health Observatory* https://www.who.int/data/gho/data/indicators/indicator-details/GHO/regional-prevalence-aafs-all-cause-deaths-(-) (2022).

[4] World Health Organization. Regional Office for Europe. *Making the WHO European Region SAFER: developments in alcohol control policies, 2010–2019*. https://apps.who.int/iris/handle/10665/340727 (2021).

[5] Nemtsov A, Neufeld M, Rehm J (2019). Are tends in alcohol consumption and cause-specific mortality in Russia between 1990 and 2017 the result of alcohol policy measures?. J. Stud. Alcohol Drugs.

[6] Neufeld M, Ferreira-Borges C, Gil A, Manthey J, Rehm J (2020). Alcohol policy has saved lives in the Russian Federation. Int. J. Drug Policy.

[7] World Health Organization. *Tackling NCDs: ‘best buys’ and other recommended interventions for the prevention and control of noncommunicable diseases*. https://apps.who.int/iris/handle/10665/259232 (2017).

[8] Chisholm D (2018). Are the ‘best buys’ for alcohol control still valid? An update on the comparative cost-effectiveness of alcohol control strategies at the global level. J. Stud. Alcohol Drugs.

[9] Anderson P, Chisholm D, Fuhr DC (2009). Effectiveness and cost-effectiveness of policies and programmes to reduce the harm caused by alcohol. Lancet Lond. Engl..

[10] Holm AL, Veerman L, Cobiac L, Ekholm O, Diderichsen F (2014). Cost-effectiveness of changes in alcohol taxation in Denmark: A modelling study. Cost Eff. Resour. Alloc. CE.

[11] Štelemėkas M (2021). Alcohol control policy measures and all-cause mortality in Lithuania: An interrupted time-series analysis. Addiction.

[12] Rehm J, Štelemėkas M, Kim KV, Zafar A, Lange S (2021). Alcohol and health in Central and Eastern European Union countries: Status quo and alcohol policy options. J. Health Inequal..

[13] de Goeij MCM (2015). How economic crises affect alcohol consumption and alcohol-related health problems: A realist systematic review. Soc. Sci. Med..

[14] Rehm J (2021). Classifying alcohol control policies with respect to expected changes in consumption and alcohol-attributable harm: The example of Lithuania, 2000–2019. Int. J. Environ. Res. Public. Health.

[15] Rehm J (2023). Classifying alcohol control policies enacted between 2000 and 2020 in Poland and the Baltic countries to model potential impact. Addiction.

[16] Tran A (2022). Predicting the impact of alcohol taxation increases on mortality: A comparison of different estimation techniques. Alcohol Alcohol. Oxf. Oxfs..

[17] Shadish WR, Cook TD, Campbell DT (2002). Experimental and quasi-experimental designs for generalized causal inference.

[18] Wagenaar AC, Tobler AL, Komro KA (2010). Effects of alcohol tax and price policies on morbidity and mortality: A systematic review. Am. J. Public Health.

[19] Statistics Estonia https://www.stat.ee/en (2022).

[20] Official Statistics Portal. Official statistics of Latvia https://stat.gov.lv/en (2022).

[21] Statistics Lithuania. State Data Agency https://www.stat.gov.lt/en (2022).

[22] Causes of Death Register. Institute of Hygiene https://www.hi.lt/en/mortality-in-lithuania.html (2022).

[23] Statistics Poland https://stat.gov.pl/en/ (2022).

[24] OECD Statistics. https://stats.oecd.org/index.aspx (2022).

[25] European Commission. Eurostat. *Revision of the European Standard Population: report of Eurostat’s task force: 2013 edition.*10.2785/11470 (2013).

[26] Rehm J (2022). Do alcohol control policies have the predicted effects on consumption? An analysis of the Baltic countries and Poland 2000–2020. Drug Alcohol Depend..

[27] Beard E (2019). Understanding and using time series analyses in addiction research. Addiction.

[28] Osborne J (2019). Notes on the use of data transformations. Pract. Assess. Res. Eval..

[29] Boisbunon A, Canu S, Fourdrinier D, Strawderman W, Wells MT (2014). Akaike’s information criterion, Cp and estimators of loss for elliptically symmetric distributions. Int. Stat. Rev..

[30] Satorra A, Bentler PM (2010). Ensuring positiveness of the scaled difference Chi-square test statistic. Psychometrika.

[31] R Core Team (2021). R: A language and environment for statistical computing.

[32] Leon DA (1997). Huge variation in Russian mortality rates 1984–94: Artefact, alcohol, or what?. Lancet Lond. Engl..

[33] Erol A, Karpyak VM (2015). Sex and gender-related differences in alcohol use and its consequences: Contemporary knowledge and future research considerations. Drug Alcohol Depend..

[34] Johnson PB, Richter L, Kleber HD, McLellan AT, Carise D (2005). Telescoping of drinking-related behaviors: Gender, racial/ethnic, and age comparisons. Subst. Use Misuse.

[35] Tucker JA, Chandler SD, Witkiewitz K (2020). Epidemiology of recovery from alcohol use disorder. Alcohol Res. Curr. Rev..

[36] OECD. *Health at a Glance 2017: OECD Indicators*. https://www.oecd-ilibrary.org/social-issues-migration-health/health-at-a-glance-2017_health_glance-2017-en (2017).

[37] Life expectancy by age and sex. *Eurostat* https://ec.europa.eu/eurostat/databrowser/view/demo_mlexpec/default/table?lang=en (2022).

[38] Stumbrys D (2020). Alcohol-related male mortality in the context of changing alcohol control policy in Lithuania 2000–2017. Drug Alcohol Rev..

[39] Zatoński W, Janik-Koncewicz K, Zatoński M (2022). Life expectancy and alcohol use health burden in Poland after 2002. J. Health Inequal..

[40] Babor, T. *et al. Alcohol: No ordinary commodity: Research and public policy, 2nd ed*. xv, 360 (Oxford University Press, 2010). 10.1093/acprof:oso/9780199551149.001.0001.

[41] Kilian C (2022). Changes in alcohol use during the COVID-19 pandemic in Europe: A meta-analysis of observational studies. Drug Alcohol Rev..

[42] Schmidt RA (2021). The early impact of COVID-19 on the incidence, prevalence, and severity of alcohol use and other drugs: A systematic review. Drug Alcohol Depend..

[43] Sohi I (2022). Changes in alcohol use during the COVID-19 pandemic and previous pandemics: A systematic review. Alcohol. Clin. Exp. Res..

[44] Karanikolos M (2013). Financial crisis, austerity, and health in Europe. The Lancet.

