# Supplementary material for: Alcohol control policies reduce all-cause mortality in Baltic Countries and Poland between 2001 and 2020
Source: Sci Rep. 2023 Apr 18;13:6326. doi: 10.1038/s41598-023-32926-5 (PMC10112307; doi:10.1038/s41598-023-32926-5)
Supplement: Supplementary file 1 — Supplementary Information. [file 41598_2023_32926_MOESM1_ESM.pdf]

SUPPLEMENTARY INFORMATION

Alcohol control policies reduce all-cause mortality in Baltic Countries and Poland between 2001 and 2020

Justina Vaitkevičiūtė[1*](ORCiD iDs 0000-0002-0710-9784), Inese Gobiņa[2,3](0000-0003-4546-491X), Kinga Janik-Koncewicz[4,5](0000-0003-4355-8007), Shannon Lange[6-8](0000-0002-8067-8932), Laura Miščikienė[1,9](0000-0003-1669-154X), Janina Petkevičienė[1,10](0000-0002-3953-8655), Ričardas Radišauskas[11,12](0000-0002-1227-2674), Rainer Reile[1,13](0000-0001-9488-887X), Mindaugas Štelemėkas[1,10](0000-0002-2040-6900), Relika Stoppel[1,14](0000-0002-2526-2117), Tadas Telksnys[1](0000-0002-4975-8467), Alexander Tran[6](0000-0001-9068-0625), Jürgen Rehm[6-8,15-20](0000-0001-5665-0385), Witold A. Zatoński[4,5](0000-0003-0718-6656), Huan Jiang[6,15](0000-0003-0898-2339)

[1] Health Research Institute, Faculty of Public Health, Lithuanian University of Health Sciences, Tilžės str. 18, 47181 Kaunas, Lithuania
[2] Department of Public Health and Epidemiology, Riga Stradiņš University, Kronvalda Boulevard 9, LV-1010 Riga, Latvia
[3] Institute of Public Health, Riga Stradiņš University, Kronvalda Boulevard 9, LV-1010 Riga, Latvia
[4] European Observatory of Health Inequalities, Calisia University, Nowy Swiat 4, 62-800 Kalisz, Poland
[5] Health Promotion Foundation, Mszczonowska 51, 05-830 Nadarzyn, Poland
[6] Institute for Mental Health Policy Research, Centre for Addiction and Mental Health, 33 Ursula Franklin Street, Toronto, Ontario, Canada, M5S 2S1
[7] Campbell Family Mental Health Research Institute, Centre for Addiction and Mental Health, 250 College St., Toronto, Ontario, Canada, M5T 1R8
[8] Department of Psychiatry, University of Toronto, 250 College Street, 8th floor, Toronto, Ontario, Canada, M5T 1R8
[9] Department of Health Management, Faculty of Public Health, Lithuanian University of Health Sciences, Tilžės str. 18, 47181 Kaunas, Lithuania
[10] Department of Preventive Medicine, Faculty of Public Health, Lithuanian University of Health Sciences, Tilžės str. 18, 47181 Kaunas, Lithuania
[11] Department of Environmental and Occupational Medicine, Faculty of Public Health, Lithuanian University of Health Sciences, Tilžės str. 18, 47181 Kaunas, Lithuania
[12] Institute of Cardiology, Lithuanian University of Health Sciences, Sukilėlių av. 15, 50162 Kaunas, Lithuania
[13] Department for Epidemiology and Biostatistics, National institute for Health Development, Hiiu 42, Tallinn 11619, Estonia
[14] University of Potsdam, Department of Economics, August-Bebel-Straße 89, 14482 Potsdam, Germany
[15] Dalla Lana School of Public Health, University of Toronto, 155 College Street, Toronto, Ontario, Canada, M5T 1P8,
[16] World Health Organization / Pan American Health Organization Collaborating Centre, Centre for Addiction and Mental Health, 33 Ursula Franklin Street, Toronto, Ontario, Canada, M5S 2S1
[17] Institute of Clinical Psychology and Psychotherapy & Center of Clinical Epidemiology and Longitudinal Studies (CELOS), Technische Universität Dresden, Chemnitzer Str. 46, 01187 Dresden, Germany
[18] Center for Interdisciplinary Addiction Research (ZIS), Department of Psychiatry and Psychotherapy, University Medical Center Hamburg-Eppendorf (UKE), Martinistraße 52, 20246 Hamburg, Germany
[19] Faculty of Medicine, Institute of Medical Science, University of Toronto, Medical Sciences Building, 1 King's College Circle, Room 2374, Toronto, Ontario, Canada, M5S 1A8
[20] Program on Substance Abuse, Public Health Agency of Catalonia, Program on Substance Abuse & WHO CC, Public Health Agency of Catalonia, 81-95 Roc Boronat St., 08005, Barcelona, Spain

Alcohol control policies

Supplementary Table S1. Date of implementation, type, and description of alcohol control policies in Estonia 2001-2020*

Date of policy implementation		Policy	Description of policy
2008	January 1	Taxation	Excise tax increases by 10% for all alcoholic beverages.
	July 1 and July 14	Taxation and Availability	Excise tax increases by 20% for all alcoholic beverages and<br>Alcoholic beverages off-premises sale prohibited between 10 p.m. and 10 a.m. next day
2010	January 1	Taxation	Excise tax increases by 10% for all alcoholic beverages.
2016	February 1	Taxation	Excise tax increases by 15% for all alcoholic beverages.
2017	February 1	Taxation	Excise tax increases by 10% for all alcoholic beverages.
	July 1	Taxation	Excise tax increases by 45% for lower alcohol content wine/fermented beverages and by 70% for beer.
2018	February 1	Taxation	Excise tax increases by 5-20% for all alcoholic beverages.

Supplementary Table S2. Date of implementation, type, and description of alcohol control policies in Latvia 2001-2020*

Date of policy implementation		Policy	Description of policy
2002	June 14	Availability	Retail sales prohibited from 10 p.m. to 8 a.m. next day.
2009	February 1	Taxation	Excise tax increases by 11.5% for beer, by 33% for wine and by 31% for spirits like vodka, brandy, whisky, liquor, rum etc.).
	July 1	Taxation	Excise tax increases by 7.9% for spirits and by 50.3% for beer.
2010	February 1	Taxation	Excise tax increases by 12.5% for wine and by 7.1% for intermediate products below 15% strength by volume (other fermented beverages which are not categorized under wine or beer, e.g., vermouth, Port wine, fruit, or berry wines etc.).
2019	March 1	Taxation	Excise tax increases by 9-12% for all alcoholic beverages.

Supplementary Table S3. Date of implementation, type, and description of alcohol control policies in Lithuania 2001-2020*

**Date of policy implementation**		**Policy**	**Description of policy**
2008	January 1	Taxation	Excise tax increases by 10% for beer and by 20% for other beverages.
		Marketing	Prohibited alcohol advertisement on tv and radio from 6 a.m. to 11 p.m.
2009	January 1	Availability	Alcoholic beverages off-premises sale prohibited from 10 p.m. to 8 a.m. next day.
2017	March 1	Taxation	Excise tax increases by 2% for spirits, by 92-94% for intermediate products, and by 111–112% for wine and beer.
2018	January 1	Availability	Alcoholic beverages off-premises sale prohibited from 8pm to 10am next day from Monday to Saturday and from 10 a.m. to 3 p.m. on Sundays.
		Marketing	Prohibited alcohol advertisement on tv, radio, and internet.
		Availability	Increasing legal minimum age to 20 years (with additional enforcement criteria such as a request an ID upon purchase of alcohol if a customer appears to be younger than 25).

Supplementary Table S4. Date of implementation, type, and description of alcohol control policies in Poland 2001-2019*

**Date of policy implementation**		**Policy**	**Description of policy**
2009	March 1	Taxation	Excise tax increases by 9% for spirits, by 14% for beer, and by 16% for wine and intermediate products.
2020	January 1	Taxation	Excise taxation increase by 10% for ethyl alcohol leading to decreased affordability **

*More details about all policies published before [1–3]

** this policy was not included in data analysis, because 2001-2019 Poland data were used.

Supplementary Table S5. Economic recession start and end dates in Lithuania, Latvia, Estonia, and Poland

**Country**	**Start of recession**	**End of recession (inclusive)**
Estonia	2008 August	2009 December
Latvia	2008 May	2010 September
Lithuania	2008 August	2009 December
Poland	N/A	N/A

References

1. Rehm J, Lange S, Gobiņa I, Janik-Koncewicz K, Miščikienė L, Reile R, et al. Classifying alcohol control policies between 2000 and 2020 in Poland and the Baltic countries to model potential impact. Addiction. 2022;[Epub ahead of print Dec 5].
2. Rehm J, Štelemėkas M, Ferreira-Borges C, Jiang H, Lange S, Neufeld M, et al. Classifying Alcohol Control Policies with Respect to Expected Changes in Consumption and Alcohol-Attributable Harm: The Example of Lithuania, 2000-2019. Int J Environ Res Public Health. 2021 Mar 2;18(5):2419.
3. Rehm J, Tran A, Gobiņa I, Janik-Koncewicz K, Jiang H, Kim KV, et al. Do alcohol control policies have the predicted effects on consumption? An analysis of the Baltic countries and Poland 2000–2020. Drug and Alcohol Dependence. 2022 Dec 1;241:109682.

Diagnostic graphs

Supplementary Figure S1. Diagnostic graphs from males' full model

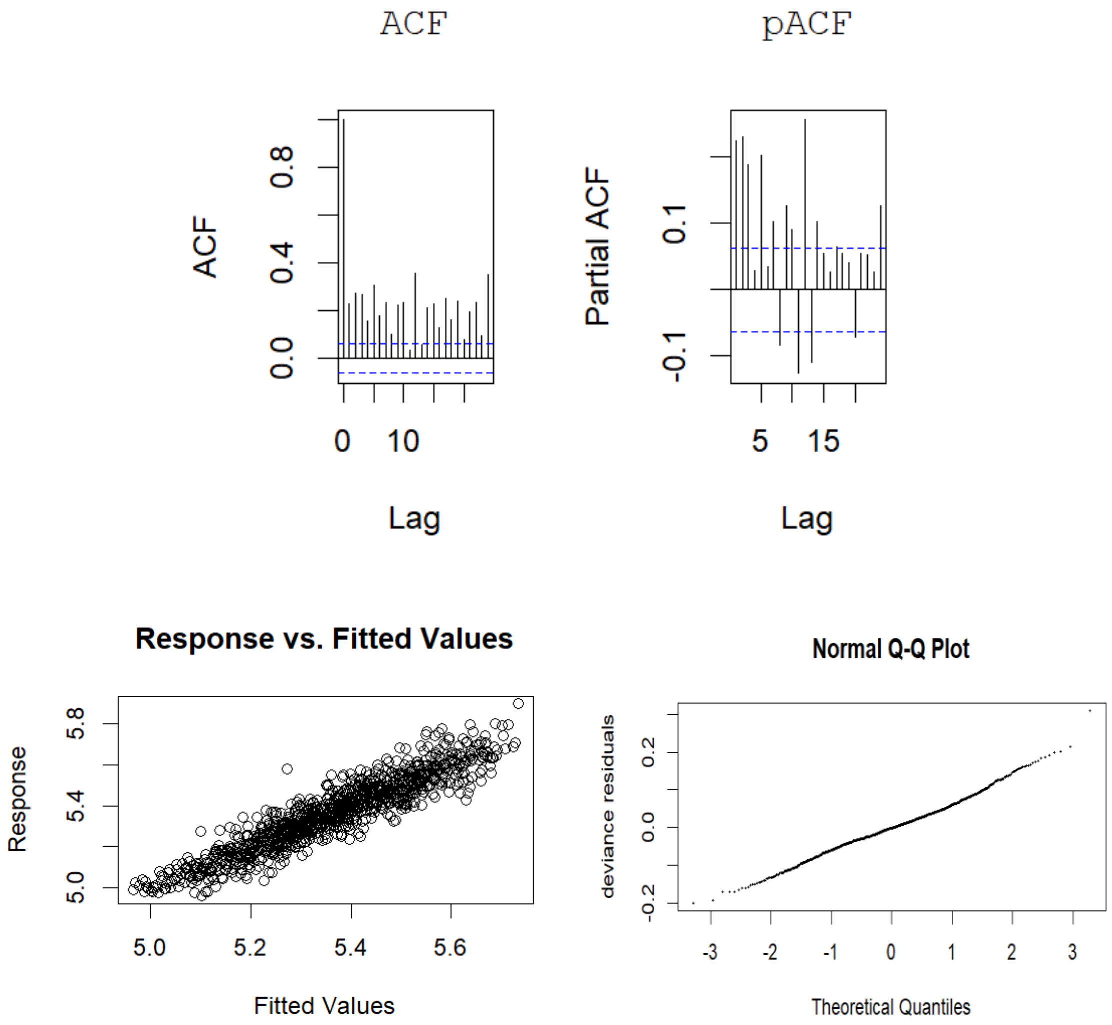

Supplementary Figure S2. Diagnostic graphs from males' reduced model

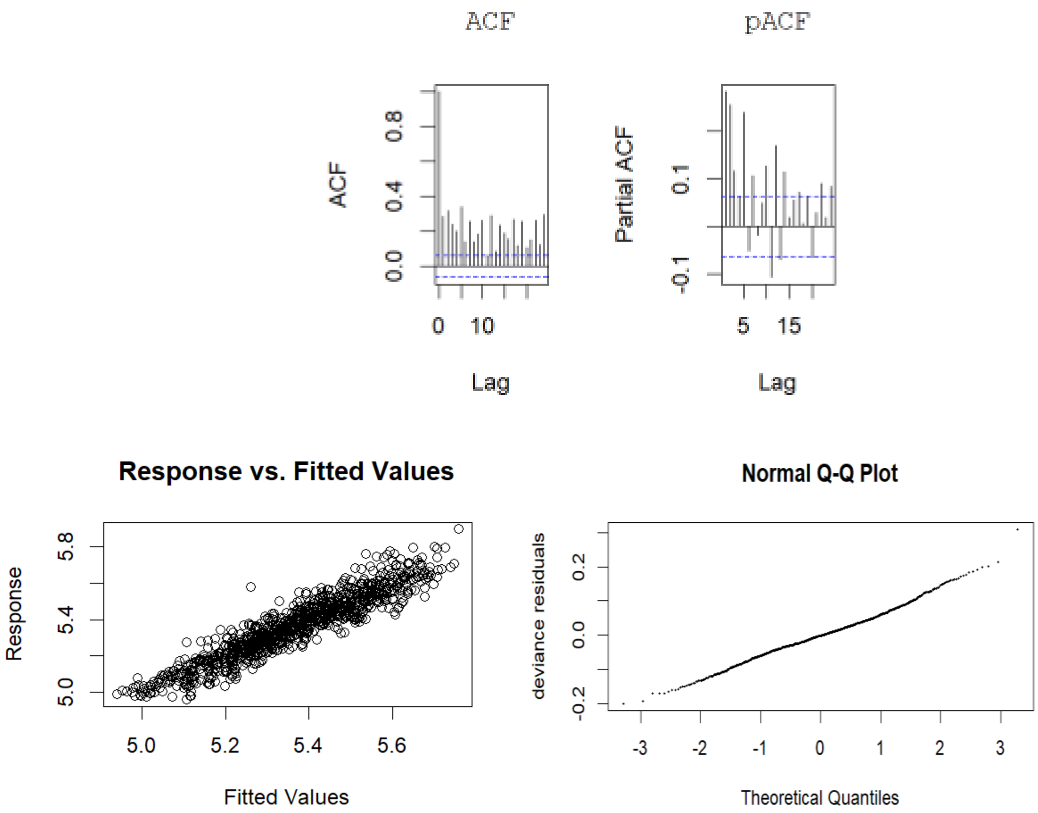

Supplementary Figure S3 Diagnostic graphs from females' full model

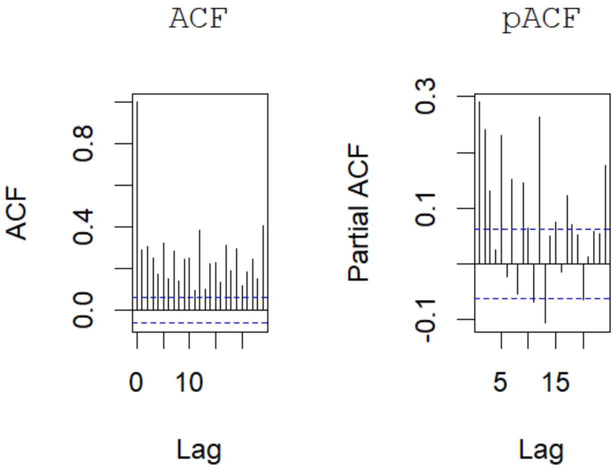

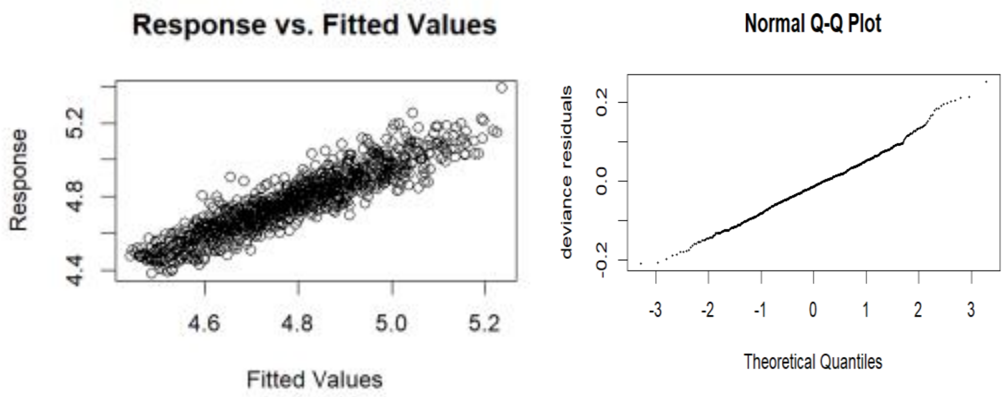

Supplementary Figure S4. Diagnostic graphs from females' reduced model

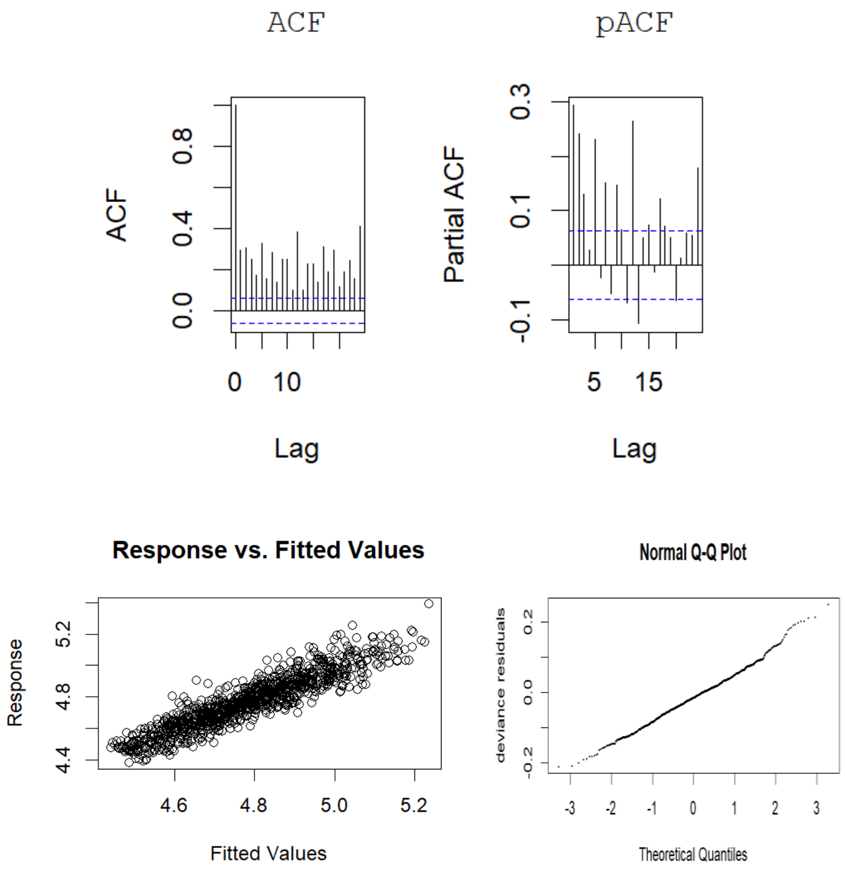

Sensitivity Analysis

Model statistics for the effects of the alcohol policies on standardized mortality rate for males, assuming policies have more durable effects: after the first year, effects decrease by 10% each year to account for inflation, higher disposable income, and adaptation to availability restrictions (Table S6).

Supplementary Table S6. Sensitivity analysis

	Full model				Reduced model			
	**Estimate**	**Std. Error**	**95% CI**	**Pr(>|t|)**	**Estimate**	**Std. Error**	**95% CI**	**Pr(>|t|)**
(Intercept)	5.3743	0.0139	(5.347,5.402)	<0.001	5.3734	0.010	(5.354,5.393)	<0.001
Time (months)	-0.0013	0.0001	(-0.001,-0.001)	<0.001	-0.0013	0.0001	(-0.001,-0.001)	<0.001
Estonia	0.1539	0.0178	(0.119,0.189)	<0.001	0.1821	0.0101	(0.162,0.202)	<0.001
Latvia	0.2627	0.0179	(0.228,0.298)	<0.001	0.2672	0.0123	(0.243,0.291)	<0.001
Lithuania	0.2141	0.0177	(0.179,0.249)	<0.001	0.2089	0.0102	(0.189,0.229)	<0.001
Policy Intervention	-0.0111	0.0139	(-0.038,0.016)	0.423	-0.017	0.0166	(-0.05,0.016)	0.3063
Recession	0.0016	0.0084	(-0.015,0.018)	0.845				
Estonia*policies	-0.0612	0.0171	(-0.095,-0.028)	4E-04	-0.0945	0.0187	(-0.131,-0.058)	<0.001
Latvia*policies	-0.0024	0.0174	(-0.037,0.032)	0.89	0.0076	0.0215	(-0.035,0.05)	0.7243
Lithuania*policies	-0.0032	0.017	(-0.037,0.03)	0.85	0.0399	0.0194	(0.002,0.078)	0.0403
	AIC = -2786				AIC = -2762			

Additional analysis

Supplementary Table S7. Causes of death (per 100,000) in the 12 months pre and post policy

Country	Sex	Period	Unintentional injuries (e.g., traffic accidents)	Intentional injury	Gastrointestinal diseases	Cancers	Ischemic heart disease	Stroke	Other cardiovascular diseases	Other causes of death	All-cause mortality rate
Estonia	Males	Pre-policy	786.75	267.80	528.62	3283.59	3268.78	894.26	2816.44	1325.35	13171.58
		Post-policy	687.86	249.68	514.88	3262.96	3107.92	834.49	2859.36	1214.11	12731.25
	Females	Pre-policy	180.43	51.77	243.08	1493.90	1817.69	635.69	1987.72	541.18	6951.46
		Post-policy	162.94	49.51	240.38	1500.60	1723.99	584.92	2027.60	492.72	6782.65
Lithuania	Males	Pre-policy	801.55	318.76	575.44	2315.66	3790.45	1208.32	896.73	1178.63	11085.54
		Post-policy	703.27	311.93	516.26	2273.58	3588.66	1175.81	866.44	1134.03	10569.97
	Females	Pre-policy	211.73	59.04	287.53	1036.19	2340.22	975.75	481.43	441.72	5833.60
		Post-policy	189.02	59.09	270.64	1034.61	2213.65	954.80	484.77	436.07	5642.65
